# Cell type of origin as well as genetic alterations contribute to breast cancer phenotypes

**DOI:** 10.18632/oncotarget.3379

**Published:** 2015-03-02

**Authors:** Divya Bhagirath, Xiangshan Zhao, William W. West, Fang Qiu, Hamid Band, Vimla Band

**Affiliations:** ^1^ Department of Genetics, Cell Biology and Anatomy, College of Medicine, University of Nebraska Medical Center, Omaha, NE 68198, USA; ^2^ Departments of Pathology and Microbiology, College of Medicine, University of Nebraska Medical Center, Omaha, NE 68198, USA; ^3^ Department of Biostatistics, College of Public Health, University of Nebraska Medical Center, Omaha, NE 68198, USA; ^4^ Biochemistry and Molecular Biology, College of Medicine, University of Nebraska Medical Center, Omaha, NE 68198, USA; ^5^ Pharmacology and Experimental Neuroscience, College of Medicine, University of Nebraska Medical Center, Omaha, NE 68198, USA; ^6^ Eppley Institute for Cancer and Allied Diseases, University of Nebraska Medical Center, Omaha, NE 68198, USA; ^7^ Fred and Pamela Buffett Cancer Center, University of Nebraska Medical Center, Omaha, NE 68198, USA

**Keywords:** transformation, stem cells, xenograft, breast cancer, metastasis

## Abstract

Breast cancer is classified into different subtypes that are associated with different patient survival outcomes, underscoring the importance of understanding the role of precursor cell and genetic alterations in determining tumor subtypes. In this study, we evaluated the oncogenic phenotype of two distinct mammary stem/progenitor cell types designated as K5^+^/K19^−^ or K5^+^/K19^+^ upon introduction of identical combinations of oncogenes-mutant H-Ras (mRas) and mutant p53 (mp53), together with either wild-type ErbB2(wtErbB2) or wild-type EGFR (wtEGFR). We examined their tumor forming and metastasis potential, using both *in-vitro* and *in-vivo* assays. Both the combinations efficiently transformed K5^+^/K19^−^ or K5^+^/K19^+^ cells. Xenograft tumors formed by these cells were histologically heterogeneous, with variable proportions of luminal, basal-like and claudin-low type components depending on the cell types and oncogene combinations. Notably, K5^+^/K19^−^ cells transformed with mRas/mp53/wtEGFR combination had a significantly longer latency for primary tumor development than other cell lines but more lung metastasis incidence than same cells expressing mRas/mp53/wtErbB2. K5^+^/K19^+^ cells exhibit shorter overall tumor latency, and high metastatic potential than K5^+^/K19^−^ cells, suggesting that these K19^+^ progenitors are more susceptible to oncogenesis and metastasis. Our results suggest that both genetic alterations and cell type of origin contribute to oncogenic phenotype of breast tumors.

## INTRODUCTION

Breast cancer is the second leading cause of cancer related deaths among women [1]. Molecular profiling of patient-derived tumors has revealed different subtypes based on gene expression signatures. The understanding of origin of various subtypes is highly important area of research considering that distinct subgroups result in significantly different outcomes, with the basal-like subtype correlating with the worst outcome, followed by claudin-low, ErbB2 over-expressing, luminal-B, normal-like and luminal-A subtype [2, 3]. The nature of genetic alterations affecting the cell may therefore play an important role in determining the pathology (i.e. latency, incidence etc.) of resulting tumors. Another important factor is the cell of origin in which the initiating oncogenic event takes place. The characteristic nature of a particular cell may determine its susceptibility towards oncogenic transformation as well as its ability to develop to primary and metastatic tumors.

To understand the importance of cell type in determining tumor phenotype, in previous study investigators isolated two distinct human mammary epithelial cells (hMECs) by culturing normal mammary tissue under different conditions, transformed the cells with an identical set of oncogenes and injected these into mammary glands of immunocompromised mice. The injected cell lines gave rise to distinct tumors depending upon their differentiation states and developed lung metastasis in a cell type dependent manner [4]. Similarly, another team of investigators showed that the origin of the transformed cell can determine the formation of tumor subtypes [5]. Both studies support the idea that breast tumor subtypes may represent malignancies of biologically distinct cell types producing distinct disease entities. However, it is still not known whether the intrinsic differences in cell lines (susceptibility to transformation) may regulate the tumor phenotype by itself or their oncogenic behaviors (transformation ability, tumor onset, incidence and metastatic capability) are also governed by the nature of genetic insults inflicted upon them. Here, we addressed these questions by subjecting two human mammary epithelial cell lines that exhibit defined differences but are cultured under identical conditions to transformation with defined oncogene combinations. Clonal cell lines corresponding to human mammary stem/progenitor cell types were previously isolated from a single healthy reduction mammoplasty specimen and immortalized using the catalytic subunit of human telomerase (hTERT). These two types of cell lines are designated as K5^+^/K19^−^ or K5^+^/K19^+^ based on cytokeratin (K) expression defining different lineage (Microarray accession no. GSE22580). Both of these cell types exhibit self-renewal and differentiate into both luminal and myoepithelial cells *in vitro* in defined medium [6, 7]. Majority of breast cancers are carcinomas and K19 positive [8, 9]. Expression of K19 can be used as prognostic marker for breast cancer [10] and presence of K19+ circulating tumor cells (CTCs) in patients before or after treatment is associated with poor disease free survival [11–13]. However, K19 positive normal mammary epithelial cells are difficult to isolate and immortalize in culture. Thus, availability of K5^+^/K19^+^ and K5^+^/K19^−^ mammary stem/progenitor cell lines generated in our laboratory provides a unique opportunity to assess their ability to serve as cells of origin for breast tumors and the impact of cell type versus oncogenes in tumor associated characteristics. Transformation of these two cell lines with different oncogene combinations was followed by extensive *in vitro* and *in vivo* analyses to demonstrate that both nature of cell type and genetic alterations contribute to the primary and metastatic behavior of tumors resulting from these cells.

## RESULTS

### *In vitro* oncogenic transformation of K5^+^/K19^−^ or K5^+^/K19^+^cells

**Figure 1:**
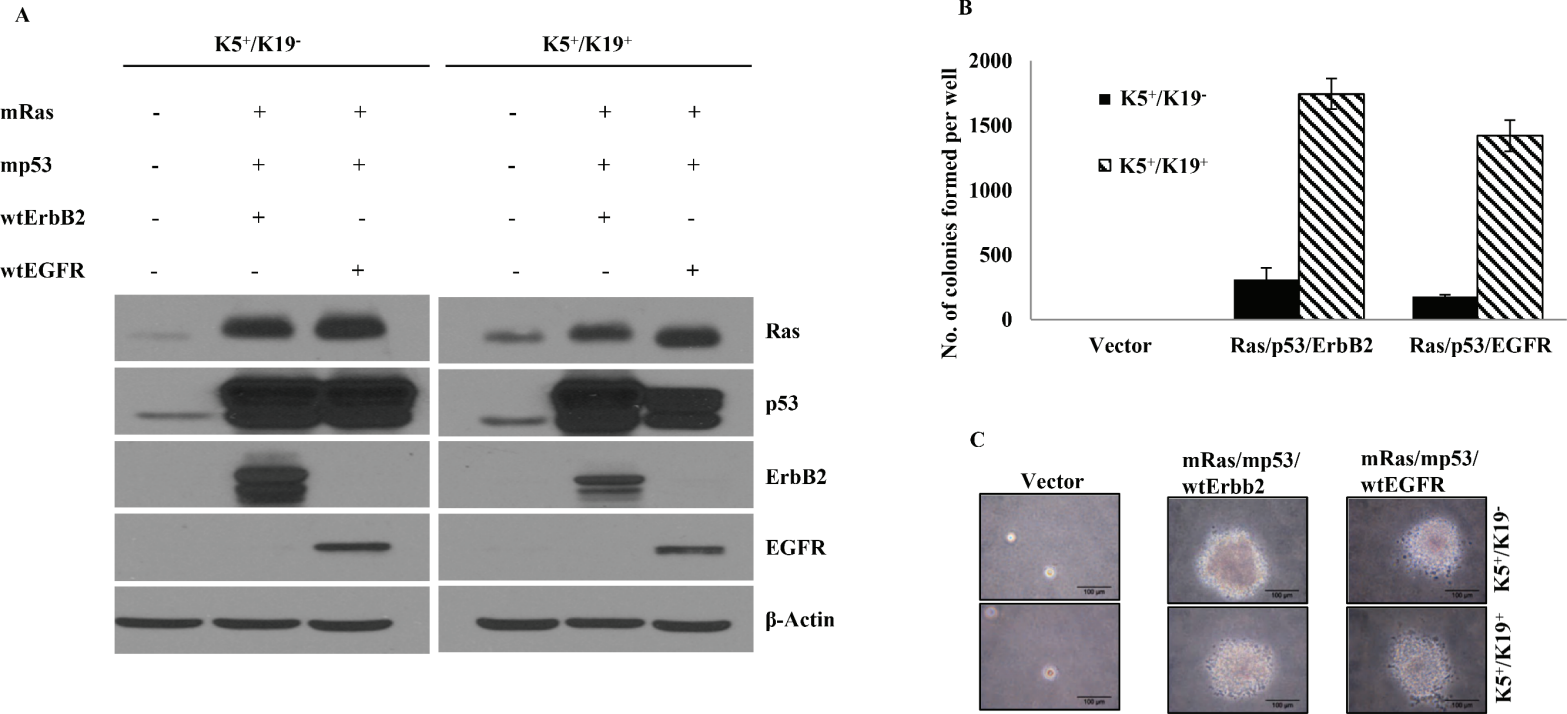
Transformation of K5^+^/K19^−^ or K5^+^/K19+ cells with different gene combination **(A)** K5^+^/K19^−^ or K5^+^/K19^+^ cell lines over-expressing mutant p53, mutant Ras, wild type ErbB2 and wild type EGFR in triple oncogene combinations were analyzed by Western Blotting. β-Actin was used as loading control. **(B)** Anchorage independent growth assay of K5^+^/K19^−^ and K5^+^/K19^+^ cells with vector or triple gene combinations. Mean ± S.D of a representative experiment done in triplicate is shown. Three independent experiments were done. **(C)** Representative images (magnification 40X) of colonies from K5^+^/K19^−^ and K5^+^/K19^+^ cells with vector or triple oncogene combination are shown here.

We have previously isolated and characterized two types of hTERT-immortalized mammary epithelial stem/progenitor cells that are designated as K5^+^/K19^−^ or K5^+^/K19^+^ based on keratin expression (Microarray accession no. GSE22580, Supplementary Table 1) [6]. We have reported previously that 100% of cells in these cell lines express designated keratins. These cell lines maintain self-renewal and are able to differentiate into both luminal and myoepithelial lineages upon culturing in defined medium [6]. We introduced mRas, mp53 along with either wtErbB2 or wtEGFR in both cell types using retroviral/lentiviral infection. The choice of mp53, wtEGFR and wtErbB2 as transforming genes was based on their wide use in the literature and their well-known occurrence in breast tumors [4, 5, 14–18]. K5^+^/K19^−^ and K5^+^/K19^+^ cells with empty vectors were used as controls in these experiments. As a first step, over-expression of various introduced genes was confirmed using western blotting (Figure 1A).

To analyze the transforming ability of exogenously introduced oncogenes and to determine susceptibility of these two cell lines to oncogene induced transformation, we performed *in vitro* soft agar assays and assessed the ability of oncogene-transduced cell lines to proliferate in an anchorage independent manner. As expected, cells expressing vectors alone failed to exhibit anchorage independent growth. K5^+^/K19^−^ and K5^+^/K19^+^ cells expressing mRas/mp53 together with either wtErbB2 or wtEGFR showed anchorage independent growth (Figure 1B, 1C). Notably, total number of colonies in K5^+^/K19^+^ cells, were significantly higher than that of colonies obtained by transformed K5^+^/K19^−^ cells (Figure 1B). These results demonstrate that *in vitro* transformation ability of a cell type is dependent on intrinsic differences within the cell lines but not the oncogene combination over-expressed by the cells.

### Transformation of K5^+^/K19^−^ or K5^+^/K19^+^cells leads to enrichment of stem cell population, and reduction in the proportion of differentiated cells

**Figure 2:**
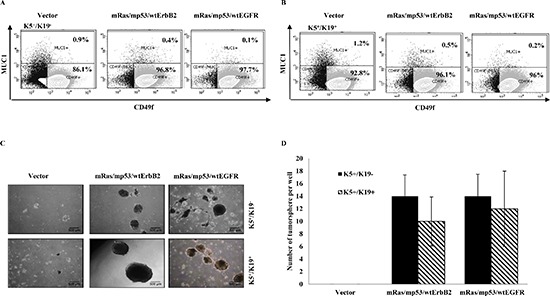
In-vitro self-renewal and differentiation of transformed K5^+^/K19^−^ or K5^+^/K19+ **(A)** and **(B)**. Control or transformed K5^+^/K19^−^ (A) or K5^+^/K19^+^ (B) cells were grown in DFCI-2 (differentiation) medium in Matrigel. Acini were trypsinized and stained with PE-Cy5 conjugated anti-CD49f and FITC conjugated anti-MUC1 and subjected to FACS analysis. **(C)** Representative images (magnification 4X) of tumorspheres from K5^+^/K19^−^ and K5^+^/K19^+^ cells with vector or triple oncogene combinations are shown. **(D)** For tumorsphere-formation assay indicated cell lines were cultured in low-attachment plates in MEGM media for 3 weeks. Spheres ≥ 200 μm were quantified. Mean +/− SD of a representative experiment done in 6 replicates is shown.

It has been shown that loss of function of the tumor suppressor p53 enhances self-renewal ability of mammary stem cells [19]. Similarly, other studies have shown that EGFR [20, 21], ErbB2 [22] or Ras [23, 24] play an important role in mammary stem cell self-renewal. We have previously shown that upon immortalization (pre-neoplastic transformation) with certain oncogenes, the stem/progenitor cell lines lose their ability to differentiate into myoepithelial cells [25]. Therefore, in this study we evaluated the impact of triple oncogene combinations on stem cell self-renewal and differentiation. We have previously shown that K5^+^/K19^−^ and K5^+^/K19^+^ mammary stem/progenitor cell lines are bi-potent stem/uncommitted progenitors and are able to differentiate into both luminal and myoepithelial cells under appropriate differentiating conditions [6]. Furthermore, when grown in 3D matrigel culture and subjected to differentiating media DFCI-2, the stem/progenitor cells are preferentially induced into luminal differentiation [25]. Notably, when we subjected K5^+^/K19^−^ and K5^+^/K19^+^ cells expressing triple oncogene combinations to differentiation using *in-vitro* 3D matrigel culture, we observed an increased CD49f+ (marker for stem cell) fraction (97% vs. 86% for K5^+^/K19^−^ and 96% vs. 92% for K5^+^/K19^+^) and decrease in MUC1+ (marker for luminal differentiation) fraction (0.4%, 0.1% vs. 0.9% for K5^+^/K19^−^ and 0.5%, 0.2% vs. 1.2% for K5^+^/K19^+^) in transformed lines vs. their controls (Figure 2A, 2B). These results indicate that oncogene-mediated transformation of mammary stem/progenitor cells reduces their ability to differentiate. Next, we assessed the ability of oncogene-transformed vs. vector control mammary stem/progenitor cell lines to form tumorspheres in ultra-low attachment plates, a commonly used assay to determine cancer stem cell self-renewal abilities [26–29]. Vector expressing cells showed formation of small spheres (< 200 μm) and were not counted (Figure 2C). Notably, cells expressing the triple combinations of oncogenes exhibited a significant increase in number of tumorsphere (> 200 μm in size) formed (Figure 2C, 2D). Both the transformed K5^+^/K19^−^ or K5^+^/K19^+^cells formed secondary tumorspheres upon re-plating (Supplementary Figure S1A) and the efficiency for tertiary tumorsphere formation was substantially enriched for cells over-expressing oncogene combination mRas/mp53/wtEGFR (Supplementary Figure S1B) demonstrating increased self-renewal capabilities of these transformed cell lines. Taken together, these results demonstrate an increase in stem cell property of triple oncogene transformed derivatives.

### Oncogene-transformed K5^+^/K19^−^ and K5^+^/K19^+^ cells produce mixed tumors in NSG mice

**Figure 3:**
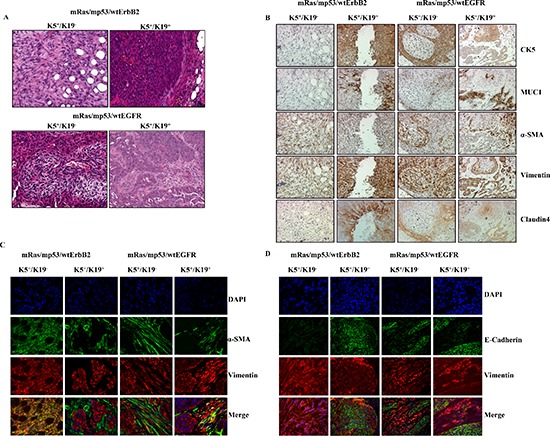
Transformed K5^+^/K19^−^ or K5^+^/K19+ cells give rise to distinct tumors **(A)** Representative images of H&E staining of tumor sections (magnification 20X) from K5^+^/K19^−^ and K5^+^/K19^+^ cells over-expressing mRas/mp53/wtErbB2 (upper panel) or mRas/mp53/wtEGFR (Lower panel). **(B)** Images from different tumors at magnification 20X. Immunohistochemical staining of tumor sections with anti-CK5 (Basal/Stem), anti-MUC1 (Luminal), anti-αSMA (Myoepithelial) anti-vimentin (Stem/myoepithelial) and claudin4 (for claudin-low) antibodies. **(C)** Representative image of tumors from K5^+^/K19^−^ orK5^+^/K19^+^ cells double immunostained with anti-αSMA (green) and anti-vimentin (red) show presence of claudin-low (SMA^+^/vimentin^+^) areas within different tumors. **(D)** Same tumor sections were double immunostained E-Cadherin (green) and vimentin (red) show presence of luminal like (E-Cadherin^+^) areas within different tumors. DAPI (blue) shows nucleus.

**Figure 4:**
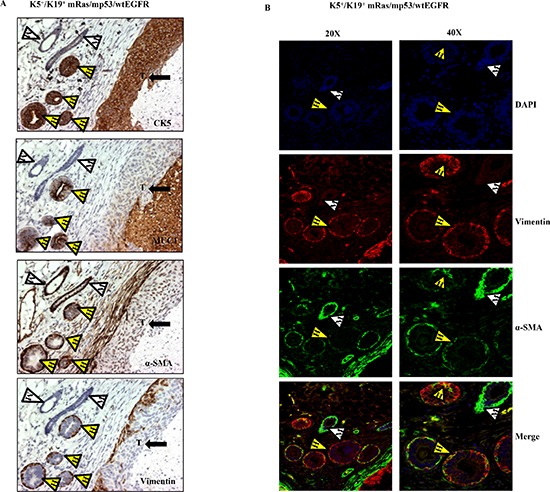
Transformed K5^+^/K19^−^ or K5^+^/K19+ cells retain stem cell characteristics in-vivo **(A)** Immunostaining with lineage specific markers of tumors and mammary duct like structures with varying degree of hyperplasia originating from K5^+^/K19^+^ cells over-expressing mRas/mp53/wtEGFR. Yellow arrowheads with H indicate human mammary ductal structures, white arrowheads with M indicate mouse mammary ducts and arrow with T shows tumor. **(B)** Immunofluorescence staining of the same tumor section with anti-vimentin (red), anti-αSMA (green) antibodies at magnifications 20X (left panel) and 40X (right panel). White arrowhead indicate mouse ductal structure, yellow arrowheads indicate co-expression in human ductal structures.

Given the *in vitro* capabilities of both cell types to exhibit anchorage independence and enhanced tumorsphere formation when transformed (Figure 1B, 1C and Figure 2D), we assessed their ability to form tumors upon orthotopic implantation in the mammary glands of immunocompromised NSG mice, as a proof of full oncogenic transformation. Triple (mRas/mp53/wtErbB2 or mRas/mp53/wtEGFR) oncogene transformed K5^+^/K19^−^ and K5^+^/K19^+^ cells were injected orthotopically in mice mammary glands. As expected, none of the mice implanted with control vector cells produced tumors while all the triple oncogene transformed K5^+^/K19^−^ and K5^+^/K19^+^ cells gave rise to tumors in mice. Histologically, tumors arising from triple oncogene combinations in K5^+^/K19^−^ and K5^+^/K19^+^ cells appeared distinct from each other. Tumors arising from transformed K5^+^/K19^−^ cells exhibited predominance of spindle-like tumor cell morphology while those arising from transformed K5^+^/K19^+^ cells resembled adenocarcinomas (Figure 3A). These differences in the tumor phenotype are consistent with previous findings that biological differences in cell types can give rise to distinct histological subtypes of tumors [4, 5]. We further observed that all tumors exhibited intra-tumoral heterogeneity as seen by the expression of various markers including MUC1 (for luminal differentiation), K5 (for stem-progenitor/basal), vimentin (for stem-progenitor/basal/myoepithelial), α-SMA (for myoepithelial) and claudin4 (for claudin-low) (Figure 3B, 3D, Supplementary Figure S2). K5^+^/K19^−^ cells over-expressing wtErbB2 combination predominantly gave rise to tumors with high claudin-low (spindle like, α-SMA^+^, vimentin^+^, claudin4^−^) and less basal (K5^+^) characteristics, whereas the same cells over-expressing wtEGFR combinations formed tumors with mixed basal, luminal and claudin-low phenotype (Figure 3A, 3B). Similarly tumors arising from K5^+^/K19^+^ cells over-expressing wtErbB2 combination showed more luminal and basal characteristics, whereas those derived by wtEGFR combination had both luminal and basal with some claudin-low components (Figure 3B-D, Supplementary Figure S2). To exclude the possibility of mouse mammary gland contribution, we used human specific vimentin antibody to confirm that the tumors were derived from the injected human cells. Vimentin antibody used here is highly specific for human cells as it neither stains mouse mammary gland (Figure 4A, 4B) nor does it detects vimentin protein in western blotting using mouse fibroblasts (Supplementary Figure S3). In order to confirm that the claudin-low component within the tumors with mixed phenotype was derived from the transformed human stem/progenitor cells, we performed double immunostaining of these tumors with human specific anti-vimentin together with anti-α-SMA, anti-E-cadherin (Figure 3C, 3D) or anti-claudin4 (Supplementary Figure S2). All tumors from transformed K5^+^/K19^−^ and K5^+^/K19^+^ stem/progenitor cells were ER-negative (data not shown) consistent with the ER-negative status of the parental normal stem/progenitor cells [6].

Interestingly, staining of tumor sections from mice injected with K5^+^/K19^+^ cells over-expressing wtEGFR combination showed both infiltrating ductal carcinoma and well-formed ductal structures with varying degrees of hyperplasia. The hyperplastic structures had a well-defined outer α-SMA-positive, vimentin positive (myoepithelial) and inner MUC1 positive (luminal) staining pattern (Figure 4A, 4B), indicative of the ability of human K5^+^/K19^+^ cells to differentiate into both myoepithelial and luminal cells *in vivo*.

### Both oncogene and cell type contribute to tumor development and progression in NSG mice

**Figure 5:**
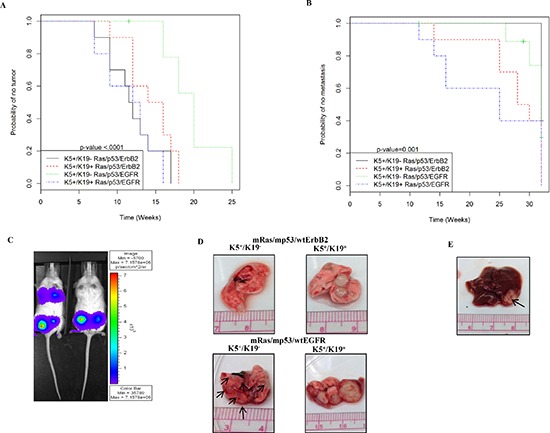
In-vivo tumor and metastasis formation from transformed K5^+^/K19^−^ or K5^+^/K19+ cells **(A)** Kaplan-Meier plot for probability of no tumor in mice injected with K5^+^/K19^−^ and K5^+^/K19^+^ cells over-expressing mRas/mp53/wtErbB2 or mRas/mp53/wtEGFR from the second experiment. **(B)** Kaplan-Meier plot for probability of no metastasis in above mentioned mice. **(C)** Representative image of mice with or without lung metastasis as shown by IVIS luciferase imaging. **(D)** Representative image of lung metastatic lesions formed by different cell types with either mRas/mp53/wtErbB2 (upper panel) or mRas/mp53/wtEGFR (Lower panel). **(E)** Representative image of liver metastasis as seen in mice injected with K5^+^/K19^−^ cells over-expressing mRas/mp53/wtEGFR.

Mice injected with cells over-expressing triple oncogene combinations developed tumors consistently (Tables 1, 2). Notably, tumors arising from mRas/mp53/wtEGFR over-expressing K5^+^/K19^−^ cell line had a statistically significant longer latency and lower tumor incidence than those arising from other cell lines tested (Figure 5A, Supplementary Table 2). The estimated time for 50% of mice to develop tumor was 20 weeks for K5+/K19^−^ cell with mRas/mp53/wtEGFR and 11.75 weeks for K5^+^/K19^−^ over-expressing mRas/mp53/wtErbB2 (Figure 5A, Supplementary Table 2). Despite the longer primary tumor latency wtEGFR combination had a higher lung metastasis incidence (Table 3, Figure 5B, 5C, Supplementary Table 3) as compared to wtErbB2 in K5^+^/K19^−^ cell line and unique liver metastasis (Figure 5E, Table 3, Supplementary Table 4). These results support the conclusion that different oncogenes can drive distinct oncogenic and metastatic potential in same cell type. On the other hand, we observed K5^+^/K19^+^ cell line over-expressing mRas/mp53/wtEGFR had a low primary tumor latency as compared to K5^+^/K19^−^ with same oncogene combination (Figure 5A, Tables 1, 2, Supplementary Table 2) and high tumor incidence (Supplementary Table 5) as compared to all other cell lines tested. More significantly, K5^+^/K19^+^ cell line over-expressing either mRas/mp53/wtErbB2 or mRas/mp53/wtEGFR had statistically significant lower lung metastasis latency (Figure 5B, Supplementary Table 6) as compared to K5^+^/K19^−^ with mRas/mp53/wtErbB2, higher lung metastasis incidence (Table 3, Supplementary Table 3) and bigger metastatic tumors (Figure 5D) as compared to K5^+^/K19^−^ cells over-expressing either of the oncogene combinations, indicating that the nature of cell type also affects the tumor and metastasis formation.

**TABLE 1:** Tumor latency for K5^+^/K19^−^ and K5^+^/K19^+^ stem/progenitor cells with different combination

Cell Line	Tumor Onset (Time first tumor appeared)	No. of mice that formed tumors (by 24 weeks)
K5^+^/K19^−^ mRas/mp53/wtErbB2	12 weeks	4/4
K5^+^/K19^+^ mRas/mp53/wtErbB2	9 weeks	4/4
K5^+^/K19^−^ mRas/mp53/wtEGFR	24 weeks	2/4
K5^+^/K19^+^ mRas/mp53/wtEGFR	12 weeks	4/4

**TABLE 2:** Tumor latency for K5^+^/K19^−^ and K5^+^/K19^+^ stem/progenitor cells with different combination

Cell Line	Tumor Onset (Time first tumor appeared)	No. of mice that formed tumors (by 16 weeks)	No. of mice that formed tumors (by 16 weeks)
K5^+^/K19^−^ mRas/mp53/wtErbB2	7 weeks	10/10	10/10
K5^+^/K19^+^ mRas/mp53/wtErbB2	9 weeks	8/10	10/10
K5^+^/K19^−^ mRas/mp53/wtEGFR	16 weeks	2/7	7/7
K5^+^/K19^+^ mRas/mp53/wtEGFR	7 weeks	10/10	10/10

**TABLE 3:** Metastasis observed with K5^+^/K19^−^ and K5^+^/K19^+^ stem/progenitor cells with different combination

Cell Line	Metastasis first appeared in mice	No. of mice that formed lung metastasis by 32 weeks	No. of mice that formed liver metastasis by 32 weeks
K5^+^/K19^−^ mRas/mp53/wtErbB2	31^st^ week	6/10	0/10
K5^+^/K19^+^ mRas/mp53/wtErbB2	25^th^ week	10/10	0/10
K5^+^/K19^−^ mRas/mp53/wtEGFR	26^th^ week	5/7	2/7
K5^+^/K19^+^ mRas/mp53/wtEGFR	11^th^ week	10/10	0/10

## DISCUSSION

Diversity in breast tumors is multifactorial. Classification of tumors in to various subtypes emphasizes the important role of genetic mutation/overexpression in characterizing a tumor [2, 17, 30]. Besides, the type of cell in which a particular genetic event occurs is another important factor that may determine the phenotype of tumors [4, 5]. While it is likely that tumor heterogeneity is of multifactorial origin, there is now wider acceptance of the idea that breast cancers exhibit a stem cell hierarchy [31–33]. Thus, one aspect of cellular diversity in tumors is thought to reflect the relative contributions to tumor mass of cells representing various points in the stem cell program. However, experimental support for the relative importance of cell types from which tumors arise versus the oncogenic events themselves has been difficult as suitable cellular models have not been available. Here, we have transformed immortal human mammary stem/progenitor cell lines with breast cancer-relevant cellular oncogenes to address these issues. Our analyses show that both the cell of origin as well as makeup of oncogenic events are important factors that shape the phenotype, oncogenicity as well as the metastatic behavior of a particular tumor.

We utilized two unique, well characterized normal human mammary stem/progenitor cell lines originating from a single donor that exhibit stable differences in keratin 19 expression as well as other genes (Microarray accession no. GSE22580, Supplementary Table 1) and are, designated as K5^+^/K19^−^ and K5^+^/K19^+.^ These cells can be propagated in culture as they have been rendered immortal with hTERT [6]. The cells remain in an undifferentiated state when grown in DFCI-1 medium but can be predictably induced to differentiate into myoepithelial and luminal lineages when cultured in differentiating media, thereby demonstrating their bi-potent nature [6]. Although majority of breast cancers are K19+ the mechanistic insights of this observation has not been addressed much in the literature. A correlative study suggested K19+ breast cancers exhibit poor prognosis [10]. Further studies have demonstrated, K19+ CTCs in breast cancer patients are associated with poor disease-free survival [11, 13]. Additionally, treatment with trastuzumab can eliminate chemotherapy resistant K19 positive CTCs and can lead to reduction in disease recurrence and increased disease-free survival, suggesting K19 positive CTCs are associated with poor disease outcome [34]. Another study demonstrated that presence of CTCs with K19+ expression during tamoxifen treatment is associated with increased risk of disease relapse [12]. Taken together, these studies underscore the importance of studying K19+ transformed breast cell lines.

Selection of transforming genes was based on a combination of relevance to human breast cancer and experimental ease. Choice of mp53, wtErbB2 or wtEGFR reflects a plethora of evidence that demonstrates a prevalent role of these in the pathogenesis of human breast cancers [14, 15, 17, 18]. For example EGFR is overexpressed in basal subtype [35], ErbB2 is overexpressed in ErbB2+ subtype [18] while p53 is known to be frequently mutated in most breast cancer subtypes [17]. While selection of mutant Ras oncogene was based on evidence from a number of investigators, that mutant active Ras functions as a potent collaborator in full transformation of human mammary epithelial cells in *in vitro*, as well as in mouse mammary orthotopic tumorigenesis models [4, 5, 16, 36]. Notably, although the frequency of Ras mutations is low in breast cancer (about 5% of total breast cancer cases) [17], activation of Ras pathway is frequently seen in breast tumors and in several breast cancer cell lines [17, 37, 38]. In addition to overexpression of upstream receptor tyrosine kinases EGFR and ErbB2 that are well known to activate Ras pathway as part of the oncogenic signaling program [37, 38], recent evidence has identified other oncogenic events that can activate the Ras pathway in breast cancer, including recurrent mutations in MAP3K1 [17, 39, 40] and mutations of RASAL2, a RasGAP gene that functions as negative regulator of Ras [41].

When we overexpressed defined combination of three oncogenes we observed an efficient anchorage independent growth in soft agar assays (Figure 1B, 1C). Consistent with the *in vitro* transformation (assessed by their ability to be anchorage independent) cells with triple oncogene combinations gave rise to tumors when orthotopically-implanted into mammary glands of immunocompromised mice. Significant differences in the onset of primary tumors and lung metastasis was observed when comparing different cell lines and different oncogene combination (Tables 1, 2, 3 Supplementary Tables 2, 3, 4, 5, 6). K5^+^/K19^+^ cells had a higher susceptibility towards transformation (Figure 1B) and had an early onset of primary tumors as well as lung metastasis as compared to K5^+^/K19^−^ (Tables 1, 2, 3, Figure 5, Supplementary Tables 2, 6) suggesting that overall these K19^+^ stem/progenitors are more prone to transformation. This may be the likely reason why more than 90% of human breast tumors are K19 positive [8, 9]. This indicates that the cell type in which tumor initiates plays an important role in development and progression of tumor. In addition we found that, presence of EGFR instead of ErbB2 in the triple oncogene combination potentially drives an early lung metastasis and give unique liver metastasis from same cellular precursor i.e K5^+^/K19^−^ (Table 3, Supplementary Table 4) suggesting the significant effect of an oncogene on pathogenicity of tumor. Most of the basal like breast tumors express or over-express EGFR and have a high propensity for metastasis. There are clinical reports that have shown an up-regulated EGFR expression in both primary and metastatic tumors [42, 43]. However the exact mechanism by which EGFR function in promoting metastasis is not very well understood. Besides the tumor latency and metastasis progression we also observed cell type dependent effect on primary tumor histology (Figure 3A). Together these data support the idea that both oncogenes and cell type in which oncogenesis was initiated contribute to key tumor traits, such as latency and incidence.

The mammary stem/progenitor cell lines utilized here are endowed with self-renewal and bipotent differentiation capabilities [6], allowing us to assess the impact of oncogenic transformation on their ability to self-renew and give rise to differentiated progeny. We present evidence that introduction of oncogenes into these stem/progenitor cell lines enhances their self-renewal capability as assayed using tumorsphere assays (Figure 2D, Supplementary Figure S1) and reduces their differentiation potential (Figure 2A, 2B). It is likely that these traits are important in the tumorigenic phenotype as we observe these only upon oncogene overexpression. This is consistent with previous findings using *in vivo* mammary tumorigenesis models that tumor cells with loss of p53 function or overexpression of ErbB2 exhibit increased self-renewal [19], and that activated Ras or over-expression of EGFR inhibit normal mammary gland differentiation [20, 24].

Tumors in mice implanted with oncogene-transformed K5^+^/K19^+^ or K5^+^/K19^−^ cell lines exhibited substantial cellular heterogeneity with respect to the components of luminal, basal and/or claudin-low like cells, which were assessed by the expression of luminal and myoepithelial cell specific markers. Oncogene-transformed derivatives of K5^+^/K19^+^ cells predominantly give luminal adenocarcinoma phenotype, whereas tumors arising from transformed K5^+^/K19^−^ cell type produced more metaplastic carcinomas (Figure 3). Thus, our results clearly show that differences in cell of origin can substantially influence the tumor phenotype, consistent with previous reports where primary human mammary epithelial cells propagated in different culture conditions were used for transformation [4].

In conclusion, using a unique set of stem/progenitor hMECs, we demonstrate that both the cell type in which oncogenic events are initiated and the nature of oncogenic events contribute to breast cancer histology, onset and incidence of primary and metastatic tumor. These unique cellular models should be highly useful to further explore the mechanisms that contribute to cellular heterogeneity in breast cancer as well as other important traits linked to cancer stem cells such as therapy resistance and poor survival.

## MATERIALS AND METHODS

**Cell lines and retroviral/lentiviral infection**. Mutant p53^R249S^ (mp53) in pLENTI-6 (purchased from Addgene) along with Invitrogen packaging vector (ViraPowerTM Lentiviral Packaging MIX) were transfected into 293FT packaging cells. Lentiviral supernatants were collected after overnight incubation in fresh DMEM media. TSA54 packaging cells were transfected with retroviral constructs, mutant H-Ras ^Q61L^ (mRas) in pBABE-hygro, wild type ErbB2 (wtErbB2) or wild type EGFR (wtEGFR) in pMSCV-puro vector or pMSCV GFP-luciferase vector (kind gift from Dr. Rakesh Singh, UNMC), together with PIK plasmid for packaging, and viral supernatants were collected (as mentioned above for lentiviral). K5^+^/K19^−^ and K5^+^/K19^+^ stem/progenitor cell lines [6] were infected with viral supernatants to generate cell lines with different gene combinations followed by their selection in DFCI-1 medium [44, 45] containing hygromycin (15 ul/ml) (for mutant H-Ras), blasticidine (15 ul/ml) (for mutant p53), puromycin (0.5 ul/ml) (for wild type ErbB2 or EGFR).

**Antibodies.** The following antibodies were used for western blotting, immunofluorescence, flow-cytometry and immunohistochemistry (IHC): Rabbit anti-human ErbB2 (sc-284), mouse anti-human p53 (DO-1) (sc-126), mouse anti-human α-smooth muscle actin (SMA) (sc-32251), mouse anti-human vimentin (sc-6260) were from Santa Cruz Biotechnology. Mouse anti-human Ras (610001), mouse anti-human EGFR (610016), mouse anti-human MUC1 (550486), rat anti-human CD49f (555734), FITC conjugated anti-CD24 (555427), PE-Cy5 conjugated anti-CD49f (551129), PE-conjugated anti-CD44 (555479), FITC conjugated anti-CD227 (MUC1–559774) and Alexa-488 conjugated E-Cadherin (560061) were from BD Bioscience. Mouse anti-claudin4 (329400) was from Invitrogen. Rabbit anti-human vimentin (clone SP20, RM-9120-S0) was from Thermo Scientific. Rabbit anti-human K5 (PRB-160P) was from Covance.

**Anchorage-independence growth assays.** 40,000 cells suspended in DFCI-1 medium containing 0.3% agarose were seeded in the top layer of each well of 6-well plates containing 0.6% agarose as a bottom layer. Each cell line was plated in triplicates. Colonies (> 60 cells) were counted after 3 weeks after crystal violet staining.

***In vitro* tumorsphere formation assays.** 40, 000 cells were plated per 2 ml in ultra-low attachment 6 well plates (Corning) in mammary epithelial growth medium (MEGM), as described previously by Dontu et al [27]. Each cell line had 6 replicates. Cells were fed with fresh medium on alternate days. Tumorspheres were counted under the microscope after 3 weeks of plating.

***In vitro* differentiation assays.** Protocol used for matrigel assay has been described previously [7, 25]. Briefly, 1 million cells suspended in DFCI-2 [44, 45] medium containing 2% matrigel were plated on P-100 dish coated with 100% reconstituted basement membrane (matrigel from BD Biosciences). After 12 days (alternate day feeding), matrigel was dissolved using dispase enzyme (BD Biosciences) at 37°C for 1 hr, cells were counted and 1 million cells were stained with FITC conjugated anti-CD227 (MUC1) and PE-Cy5 conjugated anti-CD49f and analyzed by FACS.

**Xenograft transplantation assays for primary tumor formation.** 6–8 weeks old immunodeficient NOD-SCID gamma (NSG) mice (purchased from Jackson laboratories) were injected with 1 million cells (not tagged with GFP-luciferase) in DFCI-1 medium mixed with matrigel in 1:1 proportion [4] in the fourth and ninth (contralateral) mammary glands. 4 mice were used for each combination. Tumor formation was assessed by palpation in the area of injection every week until 6 months. After six months, mice with or without tumors were sacrificed by CO_2_ inhalation followed by cervical dislocation. Tumors were excised, fixed with 10% neutral buffered formalin and processed to prepare paraffin-embedded tumor blocks that were then sectioned for IHC.

**Spontaneous metastasis formation assay.** GFP-luciferase tagged tumor cells were injected in mammary glands of NSG mice. 10 mice were used for each combination. Mammary tumors formed after xenotransplantation of GFP-luciferase tagged tumor cells were surgically removed upon reaching 250 mm^3^ tumor size. Mice were routinely assessed for luciferase activity (peritoneal injection of 30mg/ml luciferin substrate) by IVIS machine to detect any primary tumor and metastasis formation at different sites. Any subsequent tumors formed were also surgically removed and mice were assessed for metastasis formation for about 8 months.

**Immunohistochemistry.** Protocol for processing paraffin embedded tissue sections for immunostaining was essentially as described previously [6, 46]. For IHC staining, tissue sections were incubated with primary antibodies (anti-K5, anti-MUC1, anti-vimentin, anti-α-SMA or anti-claudin4) in a hydrated chamber, followed by incubation with HRP-tagged secondary IgG against the primary antibodies and subsequently processed for nuclear staining and mounting of tissues. For double-immunofluorescence staining tumor sections were processed similarly and blocked with 10% goat serum for 1 hr. These were then stained with rabbit anti-vimentin, alexa 488 conjugated anti-E-cadherin, mouse anti-α-SMA or mouse anti-claudin4 antibodies. Goat anti-rabbit alexa 594 and goat anti-mouse alexa 488 conjugated secondary antibodies (Invitrogen) were used for staining. The sections were mounted with anti-fade mounting media. Images were taken with fluorescence microscope (Zeiss axioplan 2 imaging microscope).

**Statistical Analysis.** Statistical analysis was performed to analyze the tumor and metastasis onset and incidence for each transfectant. Time to event outcomes (tumor latency and metastasis latency) were calculated using Kaplan-Meier plots and then compared among all four groups using log-rank tests. If the overall *p*-value was significant, pairwise comparisons for each pair cell types were made with Sidak's correction. Tumor onset rate at 16-week follow up, lung and liver metastasis rate at 32-week follow up were compared using Fisher's exact tests.

## SUPPLEMENTARY FIGURES AND TABLES




