# Supplementary material for: Cell type of origin as well as genetic alterations contribute to breast cancer phenotypes
Source: Oncotarget. 2015 Mar 2;6(11):9018–30. doi: 10.18632/oncotarget.3379 (PMC4496199; doi:10.18632/oncotarget.3379)
Supplement: Supplementary file 1 [file oncotarget-06-9018-s001.pdf]

SUPPLEMENTARY FIGURES AND TABLES

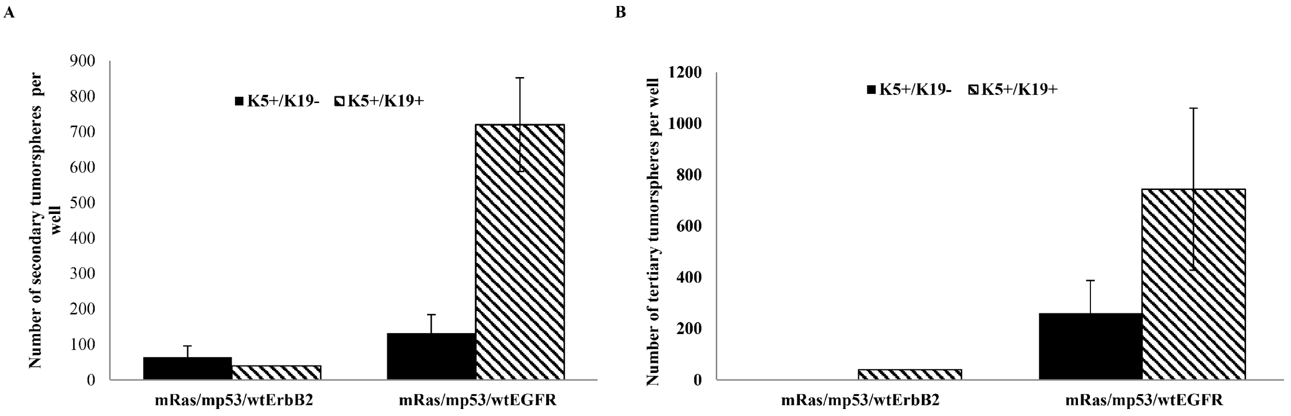

Supplementary Figure S1: *In-vitro* self-renewal and differentiation of transformed K5⁺/K19⁻ or K5⁺/K19⁺ cells. (A, B) Quantitative representation of secondary and tertiary tumorspheres formed from transformed K5⁺/K19⁻ or K5⁺/K19⁺ cells. Spheres with size bigger than > 200 µm were quantified.

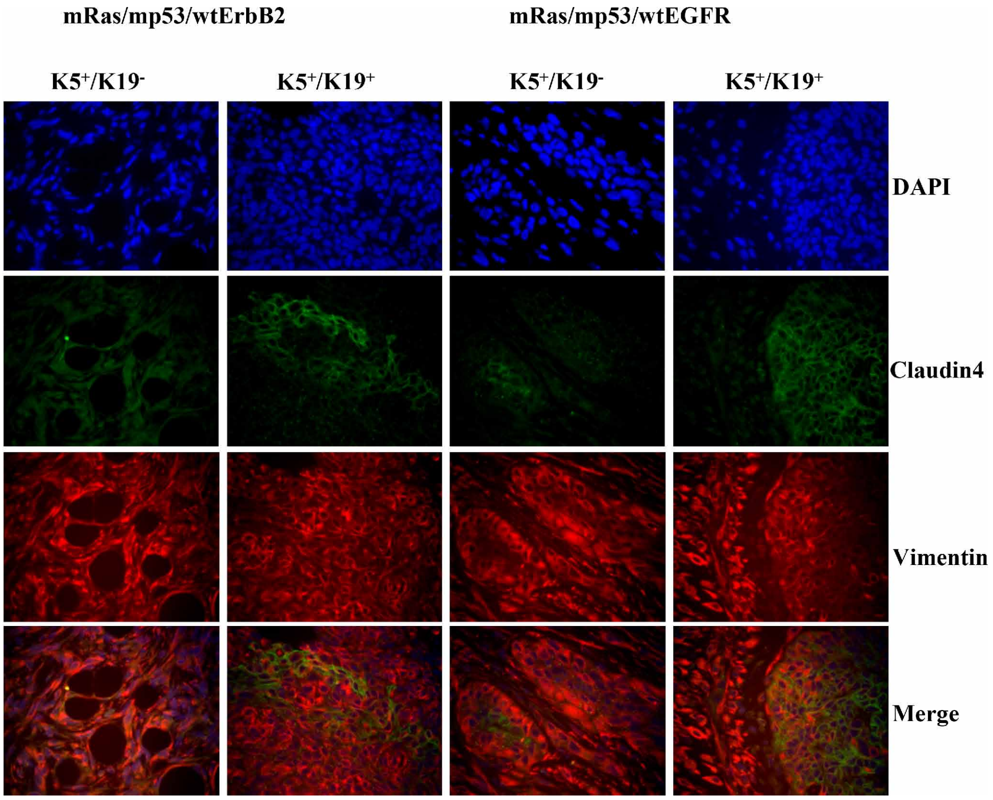

Supplementary Figure S2: Immunohistochemical staining of tumor sections with claudin4 (for claudin-low) antibody. Representative image of tumors from K5⁺/K19⁻ or K5⁺/K19⁺ cells double immunostained with anti-claudin4 (green) and anti-Vimentin (red) show presence of claudin-low (claudin4⁻/Vimentin⁺) areas within different tumors. DAPI (blue) shows nucleus.

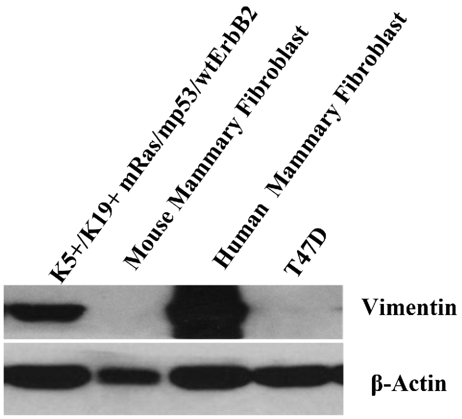

Supplementary Figure S3: Human specificity of vimentin antibody. Western blot. Cell lysates from transformed K5$^+$/K19$^+$ cells, mouse and human derived fibroblast cells and breast cancer cell line T47D were probed with human specific vimentin antibody and analyzed by Western Blotting. β-Actin was used as loading control.

Supplementary Table 1:

Supplementary Table 2: Log-rank test for time (in weeks) taken to form tumors in mice (*n* = 10 each)

Cell lines	Median survival time to first tumor (weeks)	Log-rank test overall *P*-value	Pairwise comparsion with Sidak's correction			
			K5$^+$/K19$^-$ mRas/mp53/ wtErbB2	K5$^+$/K19$^+$ mRas/mp53/ wtErbB2	K5$^+$/K19$^-$ mRas/mp53/ wtEGFR	K5$^+$/K19$^+$ mRas/mp53/ wtEGFR
K5^{+}/K19$^-$ mRas/ mp53/wtErbB2	11.75	< .0001	—	0.85	0.0002	0.99
K5$^+$/K19$^+$ mRas/ mp53/wtErbB2	15			—	0.04	0.77
K5$^+$/K19$^-$ mRas/ mp53/wtEGFR	20				—	< .0001
K5$^+$/K19$^+$ mRas/ mp53/wtEGFR	12.5					—

K5$^+$/K19$^-$ mRas/mp53/wtEGFR group had longer tumor latency compared with K5$^+$/K19$^-$ mRas/mp53/wtErbB2, K5$^+$/K19$^+$ mRas/mp53/wtErbB2, and K5$^+$/K19$^+$ mRas/mp53/wtEGFR. Sidak adjusted *p*-value: 0.0002, 0.04, and < .0001 respectively.

Supplementary Table 3: Lung metastasis rate at 32-week follow up

Variables		No metastasis *n* (%)	Lung metastasis *n* (%)	*p*-value
Cell line	K5$^+$/K19$^-$ mRas/mp53/wtErbB2	4 (40)	6 (60)	0.03
	K5$^+$/K19$^+$ mRas/mp53/wtErbB2	0 (0)	10 (100)	
	K5$^+$/K19$^-$ mRas/mp53/wtEGFR	2 (28.6)	5 (71.4)	
	K5$^+$/K19$^+$ mRas/mp53/wtEGFR	0 (0)	10 (100)	

There was indication of a difference among four groups with regard to lung metastasis rate at 32-week follow up ($p = 0.03$). The K5$^+$/K19$^-$ mRas/mp53/wtErbB2 group had lowest lung metastasis rate and K5$^+$/K19$^+$ mRas/mp53/wtErbB2 and K5$^+$/K19$^+$ mRas/mp53/wtEGFR group had the highest lung metastasis rate among four groups.

Supplementary Table 4: Liver metastasis rate at 32-week follow up

Variables		No metastasis *n* (%)	Liver metastasis *n* (%)	*p*-value
Cell line	$K5^+/K19^-$ mRas/mp53/wtErbB2	10 (100)	0 (0)	0.03
	$K5^+/K19^+$ mRas/mp53/wtErbB2	10 (100)	0 (0)	
	$K5^+/K19^-$ mRas/mp53/wtEGFR	5 (71.4)	2 (28.6)	
	$K5^+/K19^+$ mRas/mp53/wtEGFR	10 (100)	0 (0)	

There was indication of a difference among four groups with regard to liver metastasis rate at 32-week follow up ($p = 0.03$). The $K5^+/K19^-$ mRas/mp53/wtEGFR group had highest liver metastasis rate among four groups.

Supplementary Table 5: Tumor incidence rate at 16-week follow up

Variables		No tumor *n* (%)	Tumor by 16 week *n* (%)	*p*-value
Cell line	$K5^+/K19^-$ mRas/mp53/wtErbB2	2 (20)	8 (80)	0.01
	$K5^+/K19^+$ mRas/mp53/wtErbB2	3 (30)	7 (70)	
	$K5^+/K19^-$ mRas/mp53/wtEGFR	5 (71.4)	2 (28.6)	
	$K5^+/K19^+$ mRas/mp53/wtEGFR	0 (0)	10 (100)	

There was indication of a difference among four groups with regard to tumor onset rate at 16-week follow up ($p = 0.01$).The $K5^+/K19^-$ mRas/mp53/wtEGFR group had lowest tumor rate and $K5^+/K19^+$ mRas/mp53/wtEGFR group had the highest tumor rate among four groups.

Supplementary Table 6: Log-rank test for time (in weeks) taken to form metastasis in mice

Cell lines	Median survival time to first metastasis (weeks)	Log-rank test overall *P*-value	Pairwise comparsion with Sidak's correction			
			$K5^+/K19^-$ mRas/mp53/ wtErbB2	$K5^+/K19^+$ mRas/mp53/ wtErbB2	$K5^+/K19^-$ mRas/mp53/ wtEGFR	$K5^+/K19^+$ mRas/mp53/ wtEGFR
$K5^+/K19^-$ mRas/ mp53/wtErbB2	32	0.001	—	0.02	0.87	0.004
$K5^+/K19^+$ mRas/ mp53/wtErbB2	29			—	0.23	0.99
$K5^+/K19^-$ mRas/ mp53/wtEGFR	32				—	0.08
$K5^+/K19^+$ mRas/ mp53/wtEGFR	25					—

$K5^+/K19^-$mRas/mp53/wtErbB2 group took longer time to develop the first metastasis compared with $K5^+/K19^+$ mRas/mp53/wtErbB2 (median survival time in weeks: 32 vs. 29, Sidak adjusted *p*-value: 0.02), and $K5^+/K19^+$ mRas/mp53/wtEGFR (median survival time in weeks: 32 vs. 25, Sidak adjusted *p*-value: 0.004).
